# Soy Protein Isolate–Sodium Alginate Composite Particles for Stabilization of High Internal Phase Pickering Emulsions: Structural Characterization and Stabilization Mechanisms

**DOI:** 10.3390/molecules31101660

**Published:** 2026-05-14

**Authors:** Yina Yin, Yunying Li, Nan Li, Huiyun Zhang, Xinyan Peng

**Affiliations:** 1 College of Life Sciences, Yantai University, Yantai 264005, China; yyna0101@163.com (Y.Y.); 18596200343@163.com (Y.L.); linan20010908@163.com (N.L.); 2 Food and Bioengineering Department, Henan University of Science and Technology, Luoyang 471003, China; zhanghuiyun21@163.com

**Keywords:** high internal phase Pickering emulsion, replacer, sodium alginate, soy isolate protein, protein particle, interface adsorption

## Abstract

High internal phase Pickering emulsions (HIPEs) stabilized with biopolymer-based particles have sparked widespread interest due to their excellent stability and potential as fat replacements in food systems. In this study, soy protein isolate (SPI) and sodium alginate (SA) were mixed to create composite colloidal particles capable of stabilizing HIPEs with an oil phase percentage of 80%. SA significantly regulated the particle size and surface hydrophobicity of the composite particles. The optimal formulation with 1.0% SA presented a uniform particle size and desirable interfacial properties. The contact angle increased from 62.3° for pure SPI to 80.8°, which effectively improved the wettability at the oil–water interface. The interfacial protein adsorption reached a maximum of 83.7%, enabling adequate coverage of oil droplets. Low-field NMR demonstrated an increase in bound water (T_22_) from 21.893 to 30.031 (a.u.), while CLSM images confirmed the formation of compact interfacial layers. The HIPEs possessed excellent stability against heat treatment (100 °C), freeze–thaw cycling (3 cycles), high ionic strength (up to 0.6 M NaCl), and ambient storage for 30 days. These findings demonstrate that SPI-SA complexes are excellent natural stabilizers for fabricating robust, environmentally friendly HIPEs with broad prospects for functional food applications.

## 1. Introduction

Emulsions are thermodynamically unstable systems made up of two or more immiscible liquids having numerous applications in the food industry. Amphiphilic macromolecules or synthetic surfactants are commonly used to stabilize traditional emulsions due to their high solubility and interfacial activity [1]. Pickering emulsions, on the other hand, rely on the irreversible adsorption of solid particles at the oil–water interface, which increases their durability against coalescence. Inorganic particles such calcium carbonate, titanium dioxide, silica, and iron oxide have been widely used as Pickering stabilizers [2,3]. However, their proclivity to aggregate or sediment due to weak physical interactions can limit their stability and safety in food and pharmaceutical applications, creating possible health risks.

To solve these restrictions, natural, biodegradable particles—such as proteins, polysaccharides, and lipids—have emerged as a viable option. Food-grade colloidal particles, such as starch granules, cellulose, and protein–polysaccharide complexes, have been specifically developed to sustain high internal phase Pickering emulsions (HIPEs), which are defined as emulsions with an internal phase volume fraction more than 74% [4,5,6]. HIPEs have distinct features, including reduced stabilizer requirements, low environmental impact, and better physical stability [7].

Soy protein isolate (SPI) is a popular biological stabilizer due to its outstanding emulsifying capacity, nutritional value, and functional diversity [8,9]. However, native SPI alone is typically insufficient to build reliable Pickering interfaces. To address this, SPI is routinely modified or complexed with polysaccharides or polyphenols to improve interfacial behavior, particle wettability, and emulsion stability [10]. SA, an anionic polysaccharide produced from brown algae, is composed of β-D-mannuronic acid and α-L-guluronic acid residues [11]. Despite SPI-SA complexes’ intriguing potential for stabilizing HIPEs, current research is limited, particularly in terms of long-term stability and structural properties.

As a result, the purpose of this study is to look into the preparation and characterization of SPI and SA-stabilized HIPEs. The emphasis is on determining their interfacial behavior, microstructural properties, and storage stability. The findings are likely to provide theoretical insights and practical recommendations for the use of bio-based HIPEs in food, pharmaceutical, and cosmetic goods.

## 2. Results and Discussion

### 2.1. Intrinsic Fluorescence Spectra

**Figure 1:**
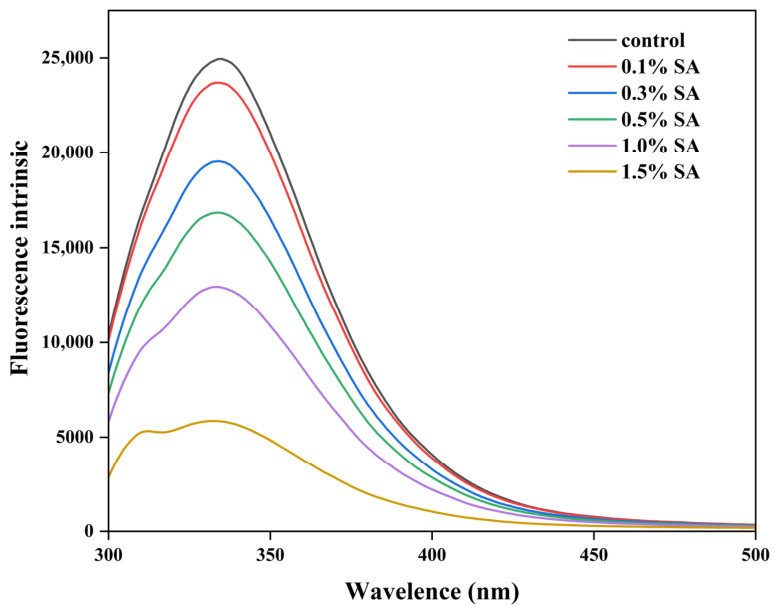
Endogenous fluorescence spectra of SPI-SA composite nanoparticles with different sodium alginate concentrations.

Endogenous fluorescence spectroscopy is used to characterise the structure of protein–polysaccharide complexes, reflecting the degree of folding of the protein tertiary structure, and thus its hydrophobicity strength, through the fluorescence intensity [12]. The fluorescence spectra of SPI and different concentrations of SA composite colloidal particles are shown in Figure 1. The maximal fluorescence intensities of all the curves are near 330 nm, which is attributed to the fact that the intrinsic fluorescence property of proteins can emit fluorescence at a specific excitation wavelength to emit fluorescence [13]. With the increase in SA concentration, the maximum emission peak intensity of SPI decreased, but the peak shape remained unchanged, showing the typical fluorescence burst phenomenon, indicating that there was an interaction between the anionic polysaccharide and SPI, and SPI and the anionic polysaccharide had been combined to form a complex [14]. The incorporation of SA increased the inter-particle spatial site resistance of SPI, which led to the change in the secondary structure of SPI, and the hydrophobic groups are exposed and the fluorescence intensity is reduced. The most significant fluorescence burst occurred in the system when the SA concentration was 1.5%, which mainly originated from the excessive concentration of polysaccharide molecules inducing a conformational change in proteins through hydrogen bonding, resulting in a sharp attenuation of the characteristic fluorescence signal intensity.

### 2.2. Three-Phase Contact Angle of Colloidal Particles

**Figure 2:**
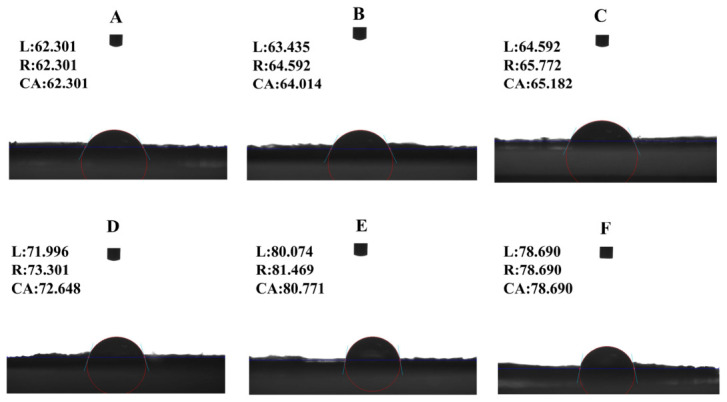
Three-phase contact angles of SPI–SA composite nanoparticles with different SA concentrations. ((**A**–**F**) represent control, 0.1% SA, 0.3% SA, 0.5% SA, 1.0% SA, and 1.5% SA, respectively).

Interfacial wettability of particles is a key factor affecting the stability of Pickering emulsions. An excessively large or small contact angle hinders particle adsorption at the oil–water interface, resulting in poor emulsion stability. Solid particles with favorable interfacial wettability can enhance the stability of Pickering emulsions, and particle wettability is generally determined by the contact angle [15]. Figure 2 visualizes the variation in the three-phase contact angle of protein–polysaccharide composite particles fabricated with different sodium alginate concentrations. As illustrated in Figure 2, the contact angle of the composite particles increased gradually with the rising dosage of sodium alginate, reaching 80.771° at 1.0 wt% SA. This value is very close to 90°, indicating a remarkable improvement in the surface hydrophobicity of the composite particles. Normally, the contact angle of particles close to 90° is conducive to the formation of strong particle adsorption at the oil–water interface, which can keep the emulsion droplets from contacting each other and avoid the instability of the emulsion [16]. However, compared with 1.0 wt% SA composite particles, the contact angle of samples containing 1.5 wt% SA decreased to 78.690°, which was lower than 90°. This further indicates that an appropriate sodium alginate concentration is critical for optimizing the stability of composite particles. In addition, it is worth noting that a smaller θ value (especially less than 90°) tends to imply that the protein complexes are more hydrophilic [17]. In conclusion, the contact angles of the composite particles increased with the addition of sodium alginate polysaccharides, and the composite particles with a concentration of 1.0 wt% SA showed the best combination of wettability and stability.

### 2.3. Particle Surface Hydrophobicity

**Figure 3:**
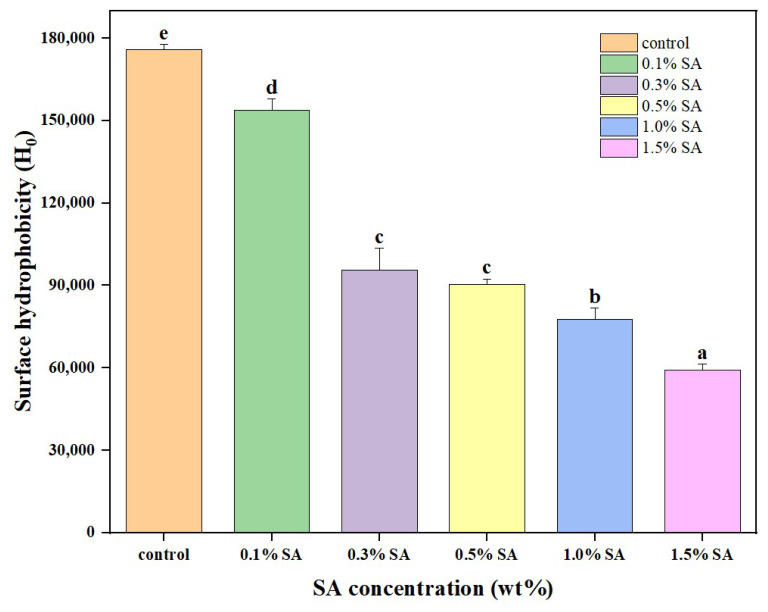
Surface hydrophobicity of SPI-SA composite nanoparticles. Different lowercase letters represent significant differences among samples (*p* < 0.05).

Surface hydrophobicity reflects protein production and its functional properties, such as solubility, gelation, and emulsification [18]. According to Figure 3, the presence of carboxylic acid groups in the SA side chains allows for the formation of hydrogen bonds with water molecules [19]. The inclusion of a large number of -OH groups through polysaccharides improves hydrogen bonding and helps to maintain the stability of the complex system [20]. Furthermore, as the amount of sodium alginate added increased, the hydrophobicity of SPI-SA decreased gradually. The surface hydrophobicity index declined fast as the sodium alginate level grew from 0.1% to 0.3%, at which point the trend of decreasing surface hydrophobicity slowed. The decrease in surface hydrophobicity could be attributed to the fact that SA is a hydrophilic molecule, and when hydrophilic sodium alginate is introduced into soybean isolate protein, the proteins’ affinity equilibrium is disrupted, the peptide chain is stretched, and the embedded hydrophilic groups are transferred to the surface [21].

### 2.4. Droplet Size Distribution of HIPE

**Figure 4:**
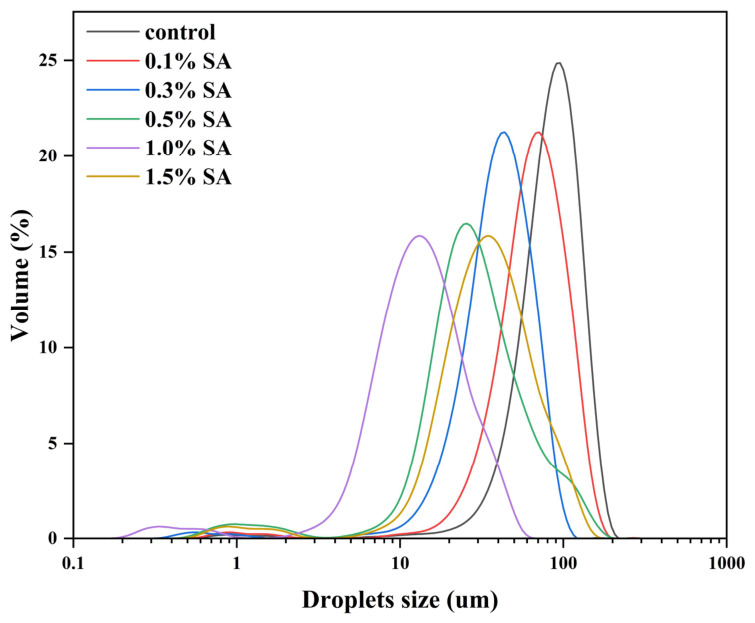
Droplet size distribution of HIPEs stabilized by SPI-SA composite particles with different SA concentrations. The control group was prepared without sodium alginate addition.

The droplet size distribution of high internal phase emulsions (HIPEs) is a key parameter for assessing emulsion stability [22]. Typically, increasing the stabilizer dosage while maintaining a constant oil content in the emulsion system results in smaller droplet diameters and improved emulsion stability [23]. As shown in Figure 4, the droplet size and size distribution of HIPEs with an 80% oil phase volume fraction, prepared using SPI-SA composite nanoparticles at varying polysaccharide concentrations, were investigated. The figure shows a negative correlation between SA concentration and emulsion droplet size, leading to smaller droplets and a more uniform size distribution. This trend indicates that SA enhances emulsification efficiency, likely by improving the surface wettability of nanoparticles during homogenization, thereby promoting increased adsorption of nanoparticles at the oil–water interface. This adsorption stabilizes smaller oil droplets, thereby contributing to a more stable emulsion. Furthermore, the increase in viscosity and the enhanced gel network structure conferred by SA further inhibit oil droplet movement and aggregation, thereby improving the overall stability of HIPEs [24]. In Figure 4, the droplet size distribution curve for the HIPE containing 1.0% SA exhibits a significantly narrower distribution compared to the other samples. This indicates that a 1.0% polysaccharide concentration promotes smaller droplet sizes and enhances HIPE stability. This is primarily due to the increased particle density at the oil–water interface. At higher concentrations of SA, the increased interfacial coverage effectively inhibits droplet aggregation and enhances the stability of the emulsion [25]. However, at a high polysaccharide concentration of 1.5% SA, non-adsorbed composite nanoparticles may tend to form depletion flocs due to strong electrostatic repulsion and thermodynamic incompatibility, resulting in an increased emulsion droplet size [26]. This suggests that higher concentrations of SA may promote droplet aggregation, thereby adversely affecting the stability and uniformity of the emulsion. In summary, 1.0% SA can aggregate within larger SPI molecular networks, thereby enhancing the hydrogen bonding interactions among them. Li et al. [27] also demonstrated that pectin reduces oil droplet size and increases the viscosity of the emulsion system. These results indicate that adding appropriate polysaccharides, such as sodium alginate (SA), can reduce droplet size in HIPPEs. In general, the droplet size characteristics of Pickering HIPEs vary greatly depending on particle type, biopolymer combination and preparation conditions [5]. Plant protein–polysaccharide composite particles are widely recognized as effective stabilizers for food-grade HIPEs, and the droplet size variation trend observed in this work is consistent with the fundamental law reported in similar SPI-based emulsion systems [22]. Moderate and homogeneous droplet size distribution is a typical structural feature of stable Pickering HIPEs, which can form a tight interfacial network and effectively resist droplet coalescence and phase separation. The regulation of droplet size by SA in this study further confirms that rational compounding of anionic polysaccharides is an effective strategy to optimize the microscopic morphology and overall stability of SPI-stabilized high internal phase emulsions.

### 2.5. HIPE Surface Protein Loading

**Figure 5:**
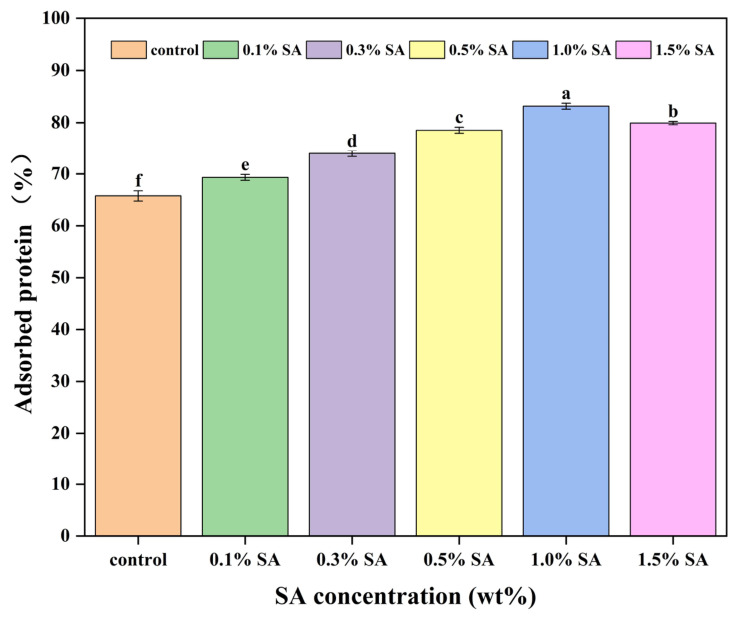
Adsorbed protein rate of HIPEs with different concentrations of sodium alginate (SA). (Different lowercase letters in the same figure indicate that there are significant differences between samples, *p* < 0.05).

Effective protein adsorption and accumulation at the oil–water interface is critical to the physical stability of emulsion systems [28]. As demonstrated in Figure 5, HIPEs stabilized with SPI-SA composite nanoparticles exhibited higher interfacial protein adsorption capacity compared with those stabilized by pure SPI, indicating that the composite nanoparticles serve as effective stabilizers. This phenomenon may be attributed to the addition of polysaccharides, which enhance electrostatic repulsion and hydrophobic interactions among interfacial proteins and thereby reduce interfacial tension. These parameters will boost the loading of composite nanoparticles at the oil–water interface for faster and more stable protein adsorption, therefore improving the protein adsorption rate [29]. SA binding to SPI resulted in efficient protein loading at the oil–water interface. The greater interfacial protein adsorption rate suggested that the protein had a high interfacial adsorption capacity, which improved the emulsion’s stability [30]. The results showed that the adsorbed protein initially increased and then reduced as the SA concentration increased. As the concentration of SA in the continuous phase increases, so does the thermodynamic incompatibility between SPI and SA, as well as the unfolding and flexibility of the SPI spatial structure. This, in turn, exposes hydrophobic and charged groups inside the SPI surface, increasing the amount of adsorbed proteins at the interface [31]. The increase in adsorbed proteins at the interface not only generates a dense interfacial coating and raises the resistance of the spatial sites, but also effectively reduces the interfacial tension between oil and water, leading to a reduction in particle size [32]. When the SA concentration was increased to 1.5%, the excess SA interfered with the interfacial adsorption of SPI due to its spatial site resistance impact and high negative charge blocking at the interface of certain protein molecules [33]. Similarly to our findings, SPI–CMC and SPI–pectin composite particles have also been demonstrated to enhance interfacial protein adsorption capacity compared to single SPI stabilizers [9,27]. For instance, Sun et al. [9] reported that SPI–carboxymethyl cellulose (CMC) complex particles stabilized HIPEs with an interfacial adsorbed protein percentage ranging from 35% to 55%, which was attributed to the synergistic interfacial adsorption between SPI and CMC, reducing protein aggregation and promoting interfacial anchoring. Similarly, Li et al. [27] found that the addition of pectin to SPI systems increased the interfacial protein adsorption efficiency by improving the surface hydrophobicity of composite particles, resulting in adsorbed protein fractions of 40–60% for HIPEs. Notably, the SPI–SA system in the present study exhibited distinct advantages in interfacial protein adsorption compared to SPI–CMC and SPI–pectin systems. The adsorbed protein rate of our SPI–SA-stabilized HIPEs (consistent with the trend in Figure 5, initially increasing and then decreasing with SA concentration) was slightly higher than that of SPI–CMC systems [9], which might be due to the stronger hydrogen bonding and electrostatic interactions between SPI and SA, leading to more stable composite particles and higher interfacial adsorption affinity. Compared with SPI–pectin systems [27], our SPI–SA system showed a more uniform interfacial protein distribution (as observed in Figure 5), which could be attributed to the appropriate molecular weight and anionic charge density of SA, avoiding excessive protein aggregation at the oil–water interface. These comparisons indicate that SPI–SA composite nanoparticles have comparable or even superior interfacial protein adsorption performance compared to other mainstream SPI–polysaccharide systems (SPI–CMC, SPI–pectin). The unique interaction between SPI and SA not only enhances interfacial protein adsorption but also improves the structural stability of HIPEs, which highlights the potential application value of SPI–SA systems in food-grade HIPE stabilization and distinguishes our findings from previous studies.

### 2.6. Stability Tests of HIPE

#### 2.6.1. Thermal Stability

**Figure 6:**
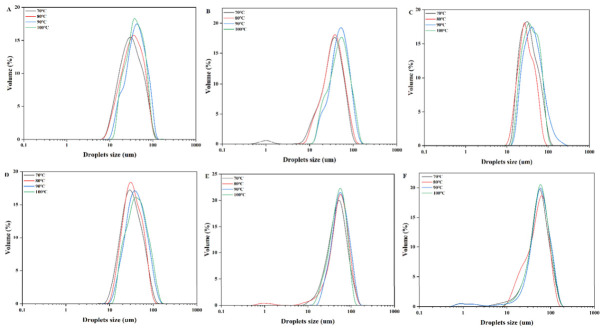
Changes in droplet size distribution of HIPEs after heat treatment at different temperatures. (**A**–**F**) represent control, 0.1% SA, 0.3% SA, 0.5% SA, 1.0% SA and 1.5% SA, respectively.

Heat treatment is one of the most common processing operations in the food industry; hence, it is essential to evaluate the thermal stability of HIPEs stabilized by composite nanoparticles with different SA concentrations. As shown in Figure 6, heating led to a decrease in oil droplet size: the average droplet size decreased from 64.66 μm to 41.27 μm at 70 °C, 69.01 μm to 44.18 μm at 80 °C, 69.71 μm to 59.56 μm at 90 °C, and 69.34 μm to 69.04 μm at 100 °C. Among all formulations, the HIPE with 1.0% SA exhibited the smallest droplet size and the slightest size variation after heating, showing outstanding thermal stability. Moderate thermal treatment facilitates molecular rearrangement of SPI–SA complexes at the oil–water interface, promoting tighter interfacial packing and disassembling loose large flocculates into finer, uniform droplets [34]. Appropriate heating further strengthens the rigidity of the interfacial network, effectively inhibiting droplet coalescence and maintaining stable microscopic morphology of HIPEs after thermal treatment Moreover, Geng et al. also discovered that the introduction of polysaccharides increased the thermal stability of emulsions due to the existence of interfacial viscoelasticity created by protein–polysaccharide complexes [35].

#### 2.6.2. Freeze–Thaw Stability of HIPE

**Figure 7:**
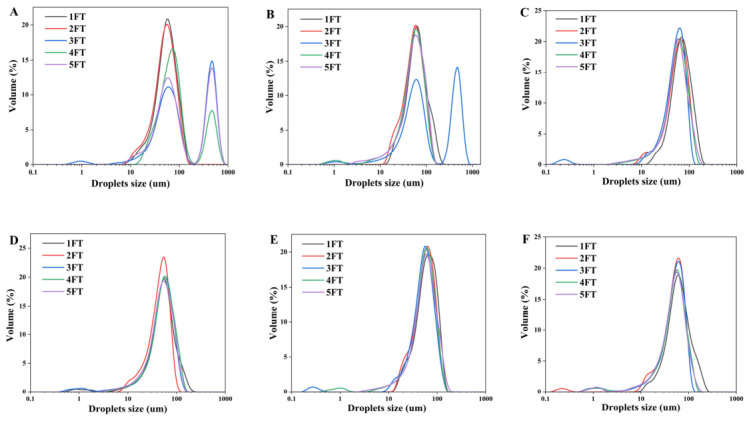
Droplet size variation in HIPEs after different freeze–thaw treatments. ((**A**–**F**) represent control, 0.1% SA, 0.3% SA, 0.5% SA, 1.0% SA, 1.5% SA; FT stands for freeze–thaw cycle, and the number in front represents the number of freeze–thaw cycles).

To extend shelf life, most meat products require frozen storage, making it necessary to evaluate HIPE stability under freeze–thaw conditions. Freeze–thaw stability is an important factor affecting the preservation of emulsion-based food products [20]. As shown in Figure 7, with the increase in polysaccharide concentration in composite nanoparticles, the oil phase precipitation of freeze–thaw-treated HIPE decreased, and the freeze–thaw resistance of the emulsion was effectively enhanced. This effect was attributed to SA forming a protective barrier of tiny macromolecules around the protein layer, reducing the potential of emulsion flocculation during the freeze–thaw cycle. Furthermore, Zhang et al. demonstrated that higher interfacial complexation significantly inhibits the development of small ice crystals, reduces the effect of freeze–thaw on HIPE structure, and maintains HIPE stability [36]. In summary, glycosylation extends the originally compact structure of SPI, improving emulsification performance. As a result, the proteoglycan particles deposit a thick adsorption layer at the oil–water interface, forming a physical barrier between the droplets. The precise distribution of emulsion droplets significantly inhibits droplet aggregation, hence increasing emulsion stability [37]. As a result, the Pickering emulsion with a 1.0% polysaccharide concentration had the best freeze–thaw stability.

#### 2.6.3. Storage Stability of HIPE

**Figure 8:**
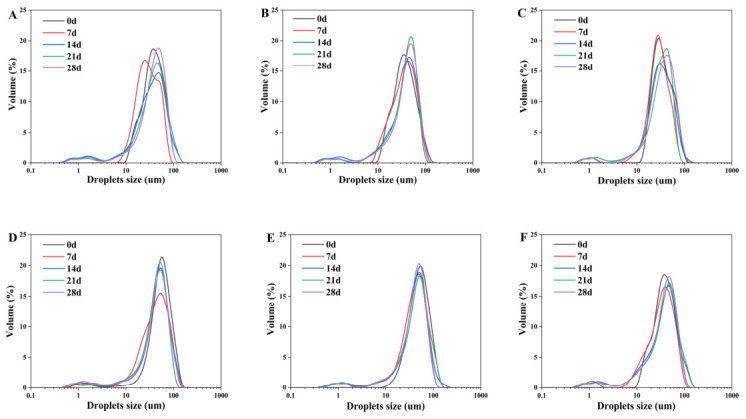
The effect of storage time on HIPE particle size distribution. ((**A**–**F**) represent control, 0.1% SA, 0.3% SA, 0.5% SA, 1.0% SA, 1.5% SA; 0 d, 7 d, 14 d, 21 d, and 28 d represent the number of days stored).

The size of emulsion droplets is influenced by the flocculation behavior of fat droplets during storage. In general, emulsion stability improves with smaller particle size and more uniform dispersion [38]. As shown in Figure 8, the particle size of HIPE emulsion of SPI group without additional polysaccharide shows instability, which is shown by the growth of particle size and the formation of bimodal peaks. As the polysaccharide concentration increased, the emulsion droplet size stability improved, and the particle size change was minimal. On the one hand, the high storage stability of the droplets against the instability of natural SPI can be attributed to the increase in electrostatic repulsion between the droplets as well as the self-assembly of SPI around the oil droplets to form a protective coating. On the other hand, the strong hydrogen bonding in the polysaccharide-protein composite nanoparticles promoted particle aggregation in the interfacial layer to form a network structure, resulting in reduced emulsion size change over time [39]. Furthermore, Feng et al. found that the droplet size of high internal phase Pickering emulsions stabilized with pea protein isolate-high methoxyl pectin complexes increased with prolonged storage time [40]. Zhou et al. used zein alcohol soluble protein-pectin hybrid particles to stabilize Pickering high internal phase emulsions, and they discovered that the HIPE remained stable for at least a month at room temperature [41]. Finally, composite nanoparticles produced with 1.0% polysaccharide content exhibited the least change in HIPE particle size and high emulsion stability.

#### 2.6.4. Ionic Strength Stability of HIPE

**Figure 9:**
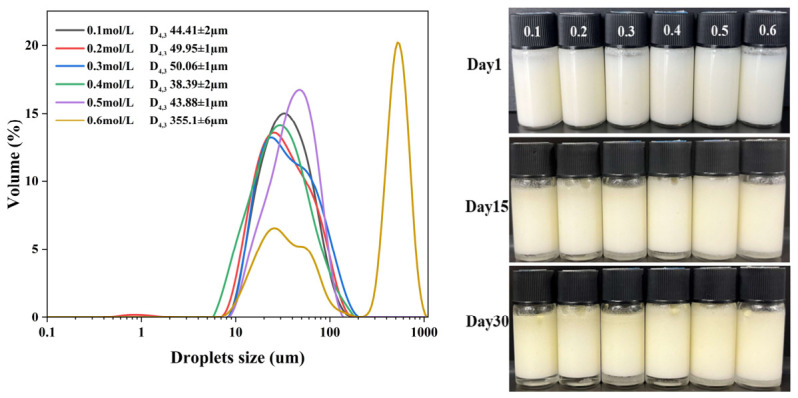
The effect of salt ion concentration on the particle size distribution and appearance of HIPE with 1.0% SA polysaccharide concentration. (0.1, 0.2, 0.3, 0.4, 0.5, 0.6 represent the salt ion concentration of 0.1~0.6 M NaCl, respectively).

Figure 9 illustrates the effect of different ionic strengths on the ionic and storage stability of HIPE prepared with 1.0% SA concentration of composite nanoparticles. Appearance observations showed that the emulsions subjected to various ionic strengths did not show any significant changes after 1 and 30 days of storage and maintained their non-flowing state. A gradual increase in emulsion droplet size was observed with increasing NaCl concentration, this increase can be attributed to the reduction in electrostatic repulsion between the emulsion droplets, which is facilitated by the presence of ionised salt forms (Na^+^ and Cl^−^). Increasing the salt ion concentration to 0.4 mol/L caused the anionic polysaccharides to pack tightly together, reducing their effective radius and thus their electrostatic repulsion; the addition of salt also reduces the attraction polysaccharides between positively charged groups of proteins and negatively charged groups of proteins, resulting in the rearrangement of the affixes on the surface of the droplets and, consequently, These findings show that the emulsion can withstand high NaCl concentrations and that the ionic strength does not jeopardize the integrity of its extremely strong crosslinked network. In addition, the addition of NaCl may reduce the mutual attraction of particles. At high ionic strengths, emulsifiers’ electrostatic repulsion has been shown to reduce or even disappear [42]. Thus, these findings indicate that spatial repulsion, rather than electrostatic repulsion, is the primary stabilizing mechanism in these emulsions.

### 2.7. Low-Field NMR

**Figure 10:**
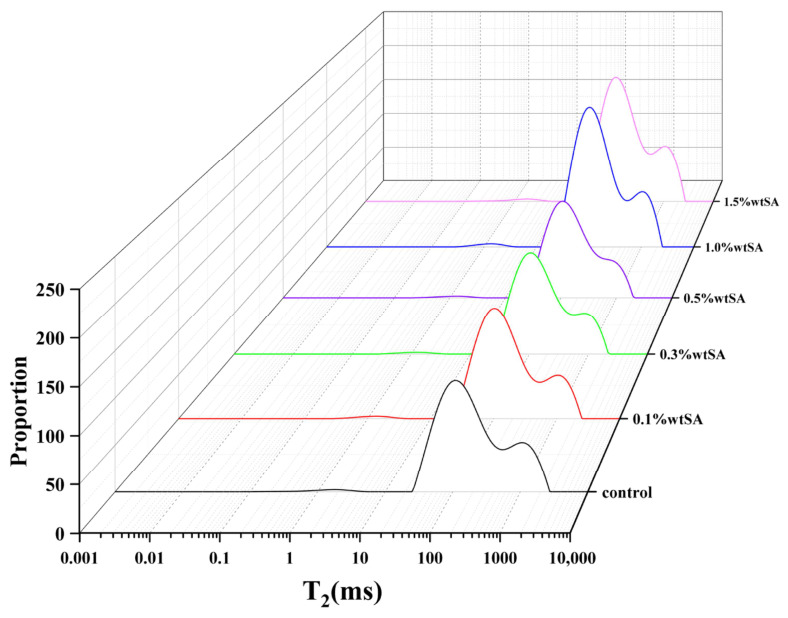
Low-field NMR relaxation characteristics of HIPEs stabilized by SPI-SA composite particles.

**Figure 11:**
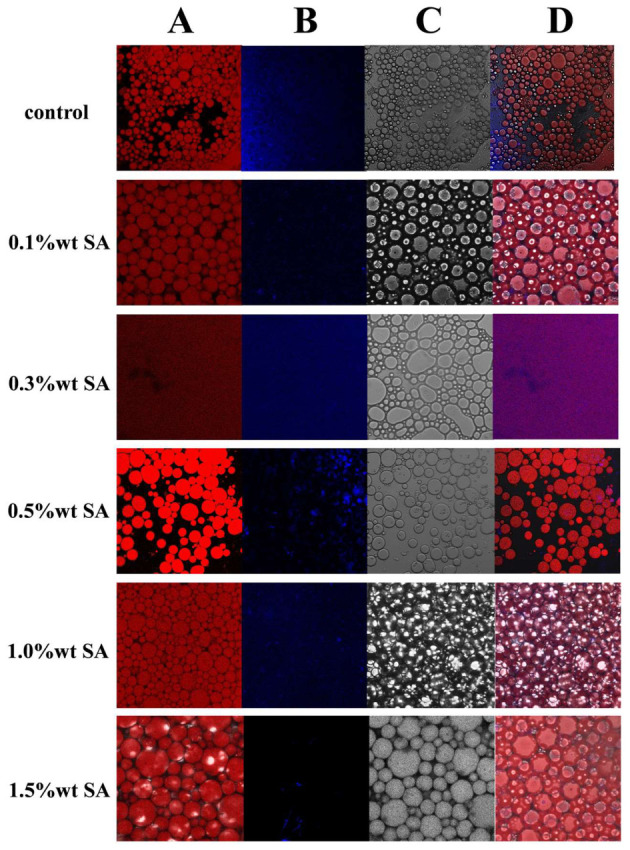
Confocal laser scanning microscopy images of HIPE, oil distribution (**A**), protein distribution (**B**), bright field shooting (**C**) and overlapping images (**D**). Scale bar: 20 µm.

LF-NMR is a rapid and non-destructive technique for characterizing the oil–water distribution in emulsions, as it provides information on hydrogen proton dynamics between water and oil phases within the sample [43]. As shown in Figure 10, with the increase in SA concentration from 0 to 1.5%, the T_22_ peak shifts leftward and the corresponding peak area increases significantly. This indicates that the addition of SA, together with the interactions between proteins and polysaccharides, restricts the mobility of oil droplets and water molecules. This result is attributable to a decrease in interfacial tension, which allows the SPI/SA complex to adsorb more efficiently on the oil droplet’s surface, limiting the chance of hydration [44]. In addition, the peak area of HIPE (T22) grew from 21,893.501 to 30,030.643, whereas HIPE (T23) declined from 10,730.539 to 7363.706. The considerable water absorption by SA resulted in the immobilization of free water molecules. Furthermore, SA absorbed water from SPI, resulting in the ‘concentration’ of SPI. This concentration event increases the non-covalent contact between SPI and SA, promoting the formation of a tight network structure that restricts the movement of oil and water molecules. As the SA addition increases from 1.0% to 1.5%, the peak area (T23) grows while the peak area (T22) falls. Excess SA may have prevented the SPI/SA network from aggregating, resulting in free water spillage, as indicated by the CLSM photos Figure 11.

### 2.8. CLSM

CLSM pictures demonstrated a similar microdistribution of oil droplets in HIPE, as illustrated in Figure 11, but emulsion samples stabilized solely by SPI exhibited higher particle sizes and were irregularly distributed. The proteins in the modified soy isolate protein emulsions with the inclusion of polysaccharides interact more with the interfacial proteins, resulting in smaller aggregates than the single soy isolate protein high internal phase emulsions [45]. It may be speculated that the formation of amide bonds and covalent cross-linking between lysine residues on SPI and sodium alginate might contribute to the generation of spherical particle aggregates, as also proposed in a previous study [46].

Beyond interphase repulsion effects, the inherent intermolecular interactions between SPI and SA also play a vital role in regulating the microstructure and overall performance of the final emulsions. As an anionic polysaccharide, sodium alginate possesses abundant hydroxyl and carboxyl groups, which can interact with polar groups and amino acid residues of SPI through hydrogen bonding and electrostatic interactions [9,13]. These non-covalent interactions facilitate the assembly of SPI–SA complexes, promote the formation of compact interfacial layers at the oil–water interface, and further construct a continuous network structure in the aqueous phase [9]. Such intermolecular associations effectively reduce droplet size, improve distribution uniformity, and enhance the structural stability of HIPE systems [13].

The higher the polysaccharide concentration, the denser the spatial network of the emulsion, and the lower the degree of droplet aggregation. The optimal performance was achieved at an SA concentration of 1.0%. With the increase in polysaccharide concentration, the emulsion droplet size gradually increased, which alleviated droplet aggregation. Meanwhile, elevated polysaccharide content strengthened the volume exclusion effect, enabling protein molecules and polysaccharides to mutually accumulate within the separated microphase [47]. Furthermore, at a polysaccharide concentration of 1.5%, the concentration of polysaccharides around the oil droplets was low due to electrostatic repulsion. The high polysaccharide concentration in the continuous phase created a significant concentration gradient from the continuous phase to the surface of the oil droplets [48]. This uneven osmotic pressure induced a depletion attraction between the droplets and the polysaccharides. Additionally, Chivero et al. demonstrated that the addition of polysaccharides leads to excessive depletion attraction, which causes the aggregation and agglomeration of oil droplets. This phenomenon results in emulsion depletion flocculation and disrupts the continuous network structure in the emulsion’s continuous phase [49].

### 2.9. DSC

**Figure 12:**
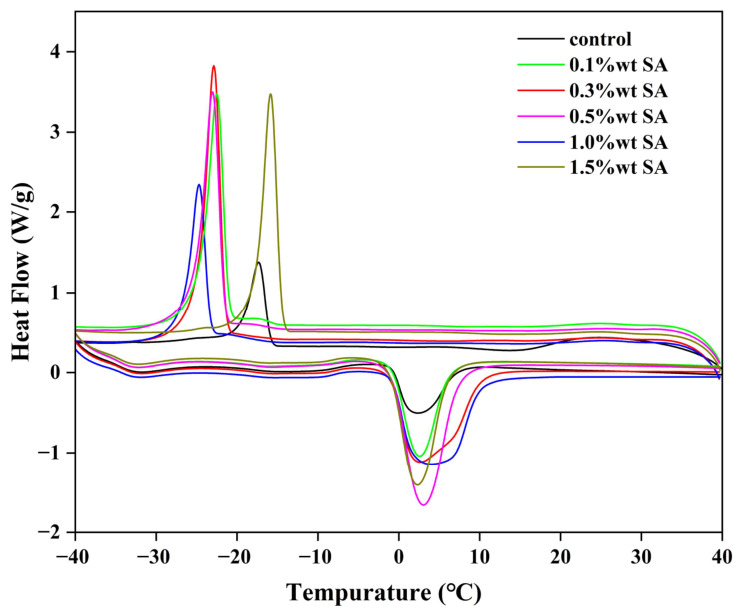
Thermal stability differences of HIPEs analyzed by differential scanning calorimetry.

Differential scanning calorimetry (DSC) is a key method for detecting structural and conformational changes in proteins. When analyzing the thermodynamic properties of macromolecules, the peak temperature obtained by DSC indirectly represents protein thermal stability, whereas the enthalpy quantifies the energy required for protein denaturation [50]. Furthermore, the shift in the heat flow peak absorption temperature provides insight into how polysaccharide–SPI complexation affects protein thermal stability. Figure 12 shows typical DSC thermal analysis curves of HIPEs stabilized by SPI–SA complexes at various SA concentrations, obtained via successive cooling and reheating processes. For all HIPEs stabilized by SPI–SA complexes with different SA contents, increasing the SA concentration gradually decreased the freezing temperature from −17.34 °C to −24.70 °C. The reduced freezing temperature indicates that increased SA content promotes the formation of a more stable internal structure in HIPEs, which is favorable for enhancing freeze–thaw stability [51].

### 2.10. Creaming Index (CI)

**Figure 13:**
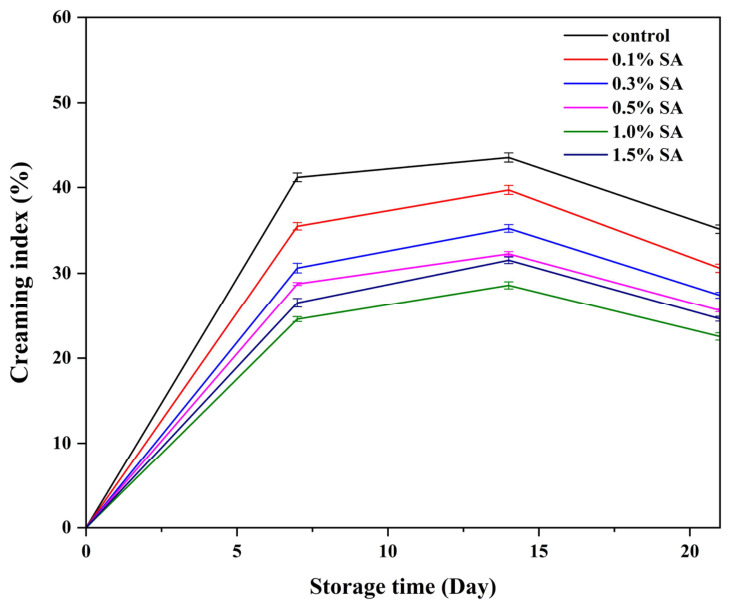
Variation in creaming index of HIPEs during storage.

The physical stability of Pickering emulsions was assessed by calculating the Creaming Index (CI), a fundamental measure for assessing the degree of separation between the lipid and aqueous phases in Pickering emulsions. As illustrated in Figure 13, HIPEs with SA concentrations ranging from 0.1% to 1.0% exhibited good long-term stability, with no signs of demulsification or oil leakage after 21 days, confirming their stability and effectiveness. This indicates that the optimal SA concentration enhanced droplet cross-linking, reduced droplet mobility, and prevented emulsification deterioration [52]. However, emulsions with SA concentrations outside this range displayed severe instability, as evidenced by increased CI values. Excess SA may lead to a low protein content in the system, destabilizing the oil–water interface and thus reducing emulsion stability [53]. This data highlights the importance of SA concentration in sustaining HIPE stability. Furthermore, Li et al. discovered that the increased physical stability of Pickering emulsions can be due to polysaccharides’ high viscosity, which effectively prevents flocculation or droplet attachment by providing spatial stability [54]. Yang et al. created Pickering emulsions with high concentrations of polysaccharide nanoparticles and remarkable physical stability. Higher nanoparticle concentrations resulted in more oil droplet coverage, which improved the durability of Pickering emulsions [55].

## 3. Materials and Methods

### 3.1. Chemicals and Materials

Linyi Shansong Biological Products Co., Ltd. (Linyi, China) provided soybean isolate protein (SPI) with a protein content of 90% or higher. Yuanye Biotechnology Co. in Shanghai, China provided sodium alginate (SA) with a purity of ≥98%. Corn oil was acquired from Jiusan Food Co., Ltd. in Harbin, China. All chemicals and reagents were analytical grade.

### 3.2. Prepare Composite Colloidal Particles

The colloidal particles used in this study were created using the method described by Yan and Jia [56], with a few modifications. Solution preparation: 2 g of SPI was dissolved in distilled water and stirred with a magnetic stirrer (EMS-30S, Changzhou Renhe Instrument Factory, Changzhou, China) for 4 h at 25 °C. The mixture was refrigerated at 4 °C overnight to thoroughly hydrate the protein, yielding a 2% (*w*/*w*) SPI stock solution. To inhibit microbial growth, 2 mM sodium azide was added to the SPI solution. Different amounts of SA powder were weighed, dissolved in 50 milliliters of distilled water, and magnetically swirled for 12 h. SA values: 0.1%, 0.3%, 0.5%, 1.0%, and 1.5% (*w*/*w*). A 10 mg/mL aqueous SPI solution served as a control. The SPI and SA solution combinations were incubated in a 95 °C water bath for 30 min to cause thermal cross-linking of SPI and SA. To obtain SPI-SA colloidal particles, the aforementioned dispersion was sheared for 2 min at 15,000 rpm using an Ultra-Turrax T18 homogenizer (IKA-Werke GmbH & Co. KG, Staufen, Germany).

### 3.3. Determination of Intrinsic Fluorescence Spectroscopy

The Fluorescence spectra were obtained using an RF-6000 fluorescence spectrophotometer (Shimadzu Enterprise Management (China) Co., Ltd., Shanghai, China; excitation: 295 nm, emission: 300–400 nm, slit width: 5 nm), as previously reported [57]. Samples were diluted with deionized water to achieve a protein concentration of 0.01 mg/mL.

### 3.4. Determination of Three-Phase Contact Angle of Colloidal Particles

SPI-SA colloidal particles were compressed into tablets at 20 MPa pressure, as described by Meng et al. [58]. The tablets were placed in a quartz cuvette with sunflower oil, and a 2-μL water droplet was deposited on their surface using a high-precision syringe. After stabilization, images were acquired using an instrument-mounted camera (Data Physics GmbH, Berlin, Germany).

### 3.5. Determination of Surface Hydrophobicity (H_0_)

Protein surface hydrophobicity (H_0_) was determined according to the method of Sun et al. [59] with slight modifications. Sample solutions were prepared in phosphate buffer (pH 7.0), and the particle dispersions were diluted to 0.003–0.015% (*w*/*v*). Then, 40 μL of 8.0 mM ANS stock solution was added to 8 mL of each diluted dispersion. After thorough mixing, the mixture was incubated in the dark for 15 min. The fluorescence intensity (FI) was recorded using an RF-6000 fluorescence spectrophotometer at an excitation wavelength of 370 nm and an emission wavelength of 470 nm. The surface hydrophobicity was evaluated by the initial slope of the relative fluorescence intensity (FI) plotted against protein concentration.

### 3.6. Preparation of High Internal Phase Pickering Emulsions

SPI aqueous dispersions with varying SA concentrations were employed as the external aqueous phase in combination with corn oil (80%), following the approach of Sun et al. with minor changes [60]. An Ultra-Turrax homogenizer (IKA T20 Basic, Staufen, Germany) was used to execute continuous shear mixing for 3 min at a speed of 13,000 rpm.

### 3.7. Determination of Emulsion Particle Size

The droplet size and distribution of HIPE were measured using a Bettersize 2000 laser particle size analyzer (Dandong Bettersize Instruments Co., Ltd., Dandong, China) [60]. An appropriate amount of sample was diluted and dispensed into the feed port. Measurement commenced when the instrument displayed an adequate absorption value. The refractive indices of corn oil and distilled water were set to 1.47 and 1.33, respectively.

### 3.8. Determination of HIPE Surface Protein Loading

Adsorbed protein percentage (AP%) was determined using the method in Ref., with some changes [61]. One milliliter of freshly made emulsion was centrifuged at 5000× *g* for five minutes. The aqueous phase of the centrifuged emulsion was collected using a syringe, and the protein concentration was calculated using Lowry’s method and BSA reagent. The AP% was computed as shown below.

$$AP(\%)=\frac{C_{O}-C_{f}}{C_{O}}\times 100\%$$

where C_O_ is the total SPI protein content, C_f_ is the protein concentration in the emulsion’s aqueous phase.

### 3.9. Determination of Stability of HIPE

#### 3.9.1. Determination of Thermal Stability

About 8 mL of the samples were transferred to a transparent glass vial and heated at 70 °C, 80 °C, 90 °C and 100 °C for 30 min. The emulsion’s appearance and microstructure were recorded after being rapidly cooled to room temperature in an ice bath [62].

#### 3.9.2. Determination of Freeze-Thawing Stability

HIPE was transferred to sample vials and frozen-thawed three times using the procedure of Chen et al. [63] with modifications. The samples were frozen at −20 °C for 24 h before thawing at 25 °C for 2 h to complete liquefaction. After three cycles, the destabilized HIPE was rehomogenized under identical conditions (as previously stated) to determine the reversibility of emulsion breakdown and reformation.

#### 3.9.3. Determination of Storage Stability

The freshly prepared HIPE was kept at room temperature for 30 days, and photographs were taken to capture the appearance of the samples [64].

#### 3.9.4. Determination of Salt Ion Stability

To observe the appearance of the emulsion, 10 mL of HIPE with 0.1–0.6 mol/L ionic concentration was obtained and stored in a glass bottle at 4 °C. The droplet size was measured and photographed.

### 3.10. Determination of Low-Field Nuclear Magnetic Resonance (LF-NMR)

HIPE was measured with a low-field nuclear magnetic resonance (LF-NMR) analyzer (NMI20-015 V-I, Niumag Co., Ltd., Shanghai, China). Freshly made emulsions were put in glass tubes (12 mm in diameter) and sealed before being scanned with an NMR probe. The emulsion’s proton density was calculated using a spin echo sequence [31], using the following parameters: FOV Read = 80 mm, FOV Phase = 80 mm, averages = 2, TR = 500 ms, TE = 20 ms, slices = 4, slice width = 2.5 mm.

### 3.11. Determination of Confocal Laser Scanning Microscopy (CLSM) Analysis

CLSM was performed using a significantly modified version of a previously described method [65]. Microscopic images of the emulsions were taken at 25 °C with a CLSM (LSM 880, Carl Zeiss AG, Heidelberg, Germany). To prepare for imaging, mix 1 mL of total polymer concentration (0.1% *w*/*v*) with 3 μL of Neil Blue solution (5 mg/mL). The emulsion sample was then mixed with a solution containing Nile Red (5 mg/mL) and Nile Blue (5 mg/mL). Nile red and Nile blue have excitation wavelengths of 488 and 633 nm, respectively.

### 3.12. Determination of Differential Scanning Calorimetry (DSC) Analysis

DSC measurements were utilized to evaluate the development and melting of ice crystals in HIPE during freezing and heating. Measurements were taken using Huang et al.’s approach [51]. The DSC studies were carried out on a TA Q200 (TA Instruments, New Castle, DE, USA), and the protocol involved cooling from 40 °C to −40 °C at 10 °C/min, annealing for 5 min, and then warming from −40 °C to 40 °C at the same rate. All HIPE samples (5–10 mg) were packed in aluminum lipid trays and examined using DSC curves.

### 3.13. Determination of Creaming Index (CI)

The creaming index (CI) of Pickering emulsions was evaluated by storing the emulsions in sealed glass tubes at room temperature for 7 weeks. The heights of the emulsion layer and the separated aqueous (slurry) layer were recorded weekly [66]. The CI was calculated as follows:

$$\mathrm{CI}\;(\%)=\frac{H_{S}}{H_{T}}\times 100\%$$

H_T_ is the total height of the emulsion in the tube, and H_S_ is the height of the separated clear aqueous layer at the bottom.

### 3.14. Statistical Analysis

Statistical computations were performed using IBM SPSS SPSS Statistics 27 (SPSS Inc., Evanston, IL, USA) and reported as mean ± standard error (SE). Tukey’s multiple comparison test was performed to identify significant differences between means (*p* < 0.05).

## 4. Conclusions

In this study, high internal phase Pickering emulsions (HIPEs) stabilized by soy protein isolate–sodium alginate (SPI–SA) composite colloidal particles were successfully prepared and systematically characterized. SA addition effectively regulated the particle size, surface wettability and hydrophobicity of SPI–SA composite particles, and significantly enhanced interfacial protein adsorption, emulsion uniformity and structural compactness in a concentration-dependent manner. The 1.0% SA group exhibited the best overall performance, with uniform droplet distribution, favorable interfacial wettability, high interfacial protein adsorption, and strong stability against heat treatment, freeze–thaw cycles and high ionic strength. CLSM and low-field NMR confirmed that 1.0% SA contributed to a compact interfacial network that restricted oil and water migration, thus improving storage stability.

These results demonstrate that SPI–SA composite particles can be used as efficient food-grade stabilizers for preparing stable HIPEs, which show promising potential as fat substitutes and lipophilic component carriers in food systems. This work provides a theoretical and practical basis for the development of bio-based, safe and ecofriendly Pickering emulsions. Nevertheless, this study still has limitations, including the lack of evaluation on cytotoxicity, sensory properties, digestion characteristics and actual food matrix applicability. Therefore, further systematic studies are needed to verify these key indicators and provide more support for the practical application and industrial development of such emulsions.

## Data Availability

The data presented in this study are available within the article.
